# Interferon Inducing Porcine Reproductive and Respiratory Syndrome Virus Vaccine Candidate Protected Piglets from HP-PRRSV Challenge and Evoke a Higher Level of Neutralizing Antibodies Response

**DOI:** 10.3390/vaccines8030490

**Published:** 2020-08-31

**Authors:** Yafei Li, Junhui Li, Sun He, Wei Zhang, Jian Cao, Xiaomei Pan, Huifen Tang, En-Min Zhou, Chunyan Wu, Yuchen Nan

**Affiliations:** 1 Department of Preventive Veterinary Medicine, College of Veterinary Medicine, Northwest A&F University, Yangling 712100, China; liyafei920521@163.com (Y.L.); 2 Scientific Observing and Experimental Station of Veterinary Pharmacology and Veterinary Biotechnology, Ministry of Agriculture, Yangling 712100, China; 3 Tecon Biology Co., Ltd., Urumqi 830000, Xinjiang, China; lijunhui@tecon-bio.com (J.L.); hesun@tecon-bio.com (S.H.); zhangwei@tecon-bio.com (W.Z.); caojian@tecon-bio.com (J.C.); panxiaomei@tecon-bio.com (X.P.); tanghuifen@tecon-bio.com (H.T.)

**Keywords:** PRRSV, modified live vaccines, HP-PRRSV, protection, neutralizing antibodies, IFN induction

## Abstract

Although widespread administration of attenuated porcine reproductive and respiratory syndrome virus (PRRSV) vaccines has been implemented since they first became commercially available two decades ago, PRRSV infection prevalence in swine herds remains high. The limited success of PRRSV vaccines is partly due to the well-established fact that a given vaccine strain confers only partial or no protection against heterologous strains. In our past work, A2MC2-P90, a novel PRRSV vaccine candidate that induced a type I IFNs response in vitro, conferred complete protection against challenge with genetically heterologous PRRSV strains. Here we assessed the ability of the PRRSV vaccine candidate A2MC2-P90 to protect piglets against the HP-PRRSV challenge and compared its efficacy to that of a licensed HP-PRRSV-specific vaccine (TJM-F92) assessed in parallel. A2MC2-P90 provided vaccinated piglets with 100% protection from a lethal challenge with extremely virulent HP-PRRSV-XJA1, while 100% mortality was observed for unvaccinated piglets by day 21 post-challenge. Notably, comparison of partial sequence (GP5) of XJA1 to A2MC2-P90 suggested there was only 88.7% homology. When comparing post-HP-PRRSV challenge responses between piglets administered A2AMC2-P90 versus those immunized with licensed vaccine TJM-F92, A2MC2-P90-vaccinated piglets rapidly developed a stronger protective humoral immune response, as evidenced by much higher titers of neutralizing antibodies, more rapid clearance of viremia and less nasal virus shedding. In conclusion, our data suggest that this novel vaccine candidate A2MC2-P90 has improved protection spectrum against heterologous HP-PRRSV strains.

## 1. Introduction

Porcine reproductive and respiratory syndrome virus (PRRSV) is a positive-sense, single-stranded, enveloped RNA virus, which belongs to the genus *Porartevirus* [1,2]. Two species within the genus *Porartevirus*, *PRRSV-1* and *PRRSV-2* [1,2], represent two genetically and antigenically distinct groups of PRRSV that share only about 60% nucleotide sequence similarity [3,4]. However, they also share nearly an identical overall disease phenotype, gross clinical signs and genomic organization [5]. PRRSV infection in vivo exhibits strict cell tropism, which is highly limited to immune cells originating from the monocyte/macrophage lineage, such as porcine alveolar macrophages (PAMs) [6,7], macrophages within peripheral lymph tissues; peritoneal macrophages in blood and bone marrow progenitor cells [8,9,10,11]. PAMs are considered to be the primary target cells of PRRSV in vivo [6,7], while certain types of primary cells, such as bone marrow-derived dendritic cells (BM-DCs) and macrophages (BM-MoCs), are also susceptible to PRRSV infection when tested in vitro [12,13]. Notably, the typical immune response in PRRSV-infected piglets is ineffective in combating the virus, resulting in persistent viremia. Additionally, infection of piglets is accompanied by signs of dysregulated immune function, such as strong suppression of innate immunity-associated cytokine release (IFN-α/β, TNF-α and IL-1β), dysregulation of Natural Killer (NK) cell function, rapid induction of non-neutralizing antibodies, delayed appearance of neutralizing antibodies, a late and low CD8^+^ T-cell response and induction of regulatory T cells [7,14,15].

The first modified live virus (MLV) vaccine against PRRSV, Ingelvac PRRS^®^ MLV (Boehringer Ingelheim), has been commercially available and widely used for more than two decades. Nevertheless, the prevalence of PRRSV infections in swine herds is still high due to the limited efficacy of this vaccine [16]. Currently, several MLV vaccines against both *PRRSV-1* and *PRRSV-2* have been licensed in various countries to combat region-specific strains circulating at each location, but such vaccines elicited only relatively weak humoral and cell-mediated immune (CMI) responses after challenge with virulent PRRSV strains when compared with other swine viral pathogens such as porcine epidemic diarrhea virus (PEDV), another member belongs to *Nidovirales* as same as PRRSV, but induces higher titer of neutralizing antibodies within two weeks after infection or immunization [17,18,19]. Meanwhile, virus challenge experiments to evaluate efficacies of PRRSV-MLVs have demonstrated that PRRSV-MLVs do confer effective, albeit late, protection against genetically homologous wild-type PRRSV strains, such as parental virulent strain for MLV. However, they only confer partial protection or no protection against cycling heterogeneous strains [20,21], which aligns with numerous reported outbreaks of atypical PRRS in previously vaccinated swine herds [22,23]. With regard to safety, it is notable that weeks-long viremia of the vaccine strain that persists in immunized piglets can lead to transmission of the vaccine virus to naive animals [20,24]. This is of particular concern if PRRSV-MLVs revert to virulence or if recombination occurs between MLVs and wild-type fields strains, with frequent occurrences of both types of events previously reported [25,26,27,28]. Therefore, a novel PRRSV-MLV vaccine with improved safety and improved cross-protection efficiency against heterogeneous PRRSV field strains is needed.

In our past work, a PRRSV strain A2MC2 (a moderately virulent strain) that uniquely induced type I IFNs release from infected MARC-145 cells and PAMs was tested for its potential as a vaccine candidate [24,29]. After in vitro attenuation of the PRRSV-A2MC2 via 90 serial passages in MARC-145 cells [30], the resulting strain A2MC2-P90 retained the ability to induce IFNs in cell culture and was selected as a vaccine candidate for animal testing [30]. Subsequently it was found that immunization with A2MC2-P90 protected piglets against challenge with VR-2385 (which shares 92.3% nucleic acid identity with A2MC2-P90) and also reduced nasal shedding of highly virulent PRRSV strain MN184 (with 84.5% nucleic acid identity to A2MC2-P90) [31]. These results highlight A2MC2-P90’s potential as a better MLV candidate with improved cross-protection efficiency for the prevention of both PRRSV infection and transmission. However, it is unknown whether A2MC2-P90 would be capable of protecting piglets from infection after challenge with highly pathogenic PRRSV (HP-PRRSV), which is highly prevalent in China.

In the current study, we systematically investigated the protective efficacy of the vaccine candidate PRRSV-A2MC2-P90 against HP-PRRSV challenge and compared the results to parallel results obtained for a licensed HP-PRRSV-specific MLV strain TJM-F92. Our results demonstrated that A2MC2-P90 vaccination of piglets conferred 100% protection against PRRSV infection after challenge with an extremely virulent HP-PRRSV strain XJA1 (HP-PRRSV-XJA1, 88.7% sequence identity to PRRSV-A2MC2-P90 based on alignment of GP5 sequence between two strains). By contrast, HP-PRRSV-XJA1 inoculation of non-vaccinated piglets led to 100% mortality by 21 dpc, while HP-PRRSV-XJA1 challenge of A2MC2-P90-vaccinated piglets elicited a rapid humoral immune response characterized by much higher titers of neutralizing antibodies, earlier clearance of viremia and less virus shedding than observed for piglets vaccinated with MLV TJM-F92. In conclusion, our data suggest that A2MC2-P90 is a novel vaccine candidate with broad improved protection spectrum against heterogeneous PRRSV strains.

## 2. Materials and Methods

### 2.1. Cells, Viruses, Chemicals, Plasmids and Interferon Bioassay

MARC-145 (simian kidney epithelial cells line derived from MA-104) and VERO cells were purchased from the China Center for Type Culture Collection (CCTCC, Wuhan, China) and cultured in Dulbecco’s modified Eagle medium (Biological Industries, Beit-Haemek, Israel) supplemented with 10% fetal bovine serum (FBS, Biological Industries), 100 U/mL of penicillin and 100 mg/mL of streptomycin. All cultures were incubated at 37 °C in a humidified atmosphere containing 5% CO_2_.

The full-genome cDNA sequence of interferon-inducing PRRSV vaccine candidate A2MC2-P90 (GenBank: KU318406) was artificially synthesized by Tsingke Bio-Tech (Beijing, China) and ligated into the pBeloBAC11 vector to construct a DNA-launched infectious clone using methods reported previously [32,33]. PRRSV A2MC2-P90 virus was recovered from the infectious clone pBAC-A2MC2-P90 by transfecting the plasmid into CRL-2843^CD163^ cells, PK-15^CD163^ cells and HEK-293T^CD163^ cells using the FuGENE^®^ HD Transfection Reagent (Promega, Madison, WI, USA) according to the manufacturer’s instructions. Stably cell lines CRL-2843^CD163^, PK-15^CD163^ and HEK-293T^CD163^ cells were generated by introducing porcine CD163-coding sequences into their parental cell lines via lentiviral vector-based transduction and were previously reported [34,35,36]. All transfected and transduced cells were maintained under the same aforementioned conditions used to maintain MARC-145 cells.

The commercial PRRSV-TJM-F92 MLV vaccine strain (TECON Biotech Co., Ltd., Urumqi, Xinjiang, China) was assessed in parallel with PRRSV-A2MC2-P90 to allow comparisons to be made between strains. The extremely virulent HP-PRRSV strain XJA1 (HP-PRRSV-XJA1), previously isolated from a farm near Urumqi reporting a PRRS outbreak with 100% mortality in piglets, was used as the challenge PRRSV strain. Although the full genomic sequence of HP-PRRSV-XJA1 is not yet available, an partial genome sequencing (GP5) for HP-PRRSV-XJA1 has confirmed that it shares very high homology (more than 99%) with HP-PRRSV-JXA1 strain (GenBank: EF112445.1) but shares only 88.7% identity to A2MC2-P90. All PRRSV isolates were propagated and titrated in MARC-145 cells.

For Interferon bioassay, cell culture supernatants of MARC-145 infected with PRRSV-A2MC2-P90 for 24 h were collected and used to treat VERO cells (non-permissive for PRRSV) for 24 h with 2 folds gradient dilutions. Next, supernatant-treated VERO cells were further infected with an interferon-sensitive Newcastle disease virus (NDV) carrying a green fluorescent protein (GFP) reporter gene at a multiplicity of infection (MOI) of 0.1 as previously described [30]. After 24 h, NDV-GFP-infected VERO cells were observed under a Leica DM1000 fluorescence microscope (Leica Microsystems, Wetzlar, Germany) for evaluation of GFP-positive cells. VERO cells treated with 10 ng recombinant human IFN-α2b (Genscript, Nanjing, China) was included as the positive control. Recombinant NDV-GFP (LaSota strain carrying virulence F cleavage sites and enhanced GFP as the reporter gene) was a gift of Dr. Sa Xiao of Northwest A&F University.

### 2.2. Ethics Statement and Animal Studies

The animal protocol was reviewed and approved by the Animal Welfare Committee of Northwest A&F University. All animals were monitored on a daily basis for any clinical signs. Four-weeks-old piglets were obtained from a PRRSV-free pig farm near Urumqi, Xinjiang and further screened to detect CSFV, PRRSV, PCV2 and ASFV along with corresponding antibodies. Only piglets (*n* = 16) negative for all examined pathogens and antibodies against PRRSV and ASFV were selected for this study. Piglets were randomly divided into four groups (*n* = 4) and housed in separate rooms. Details of piglet groupings are provided in Table 1.

### 2.3. Vaccination and HP-PRRSV Challenge

Two groups of piglets were intramuscularly immunized with PRRSV-A2MC2-P90 or PRRSV-TJM-F92 using 1 mL of viral stock (1.0 × 10^6^ TCID_50_/mL) per piglet. For non-immunized piglets, an equal volume of PBS was administered. Three weeks after immunization, serum samples from all piglets were harvested and subjected to ELISA to confirm induction of PRRSV-specific antibodies. Vaccinated piglets and unvaccinated controls were challenged via inoculation with the HP-PRRSV-XJA1 strain then blood and nasal swab samples were collected at indicated times and rectal temperatures and deaths were recorded on a daily basis. All surviving piglets were necropsied at 21 days post-challenge (dpc) for pathological examination.

### 2.4. Pathological Examination

To evaluate the protective efficacy of the PRRSV-A2MC2-P90 vaccine candidate against the HP-PRRSV challenge, lungs of challenged vaccinated piglets and challenged unvaccinated piglets were examined for gross pathological changes immediately after death or after euthanization of the remaining survivors at 21 dpc. A previously described gross lung lesion score system was used to quantify pathological changes [37]. Briefly, each lung lobe (including anterior, middle and caudal parts of the ventral and dorsal aspect and accessory lobe) was separately assigned a number of points (100 points in total). Based on pathological changes observed for each lobe part, a score was generated for each piglet that reflected the overall percentage of the entire lung that exhibited markedly visible signs of pneumonia. Meanwhile, lung tissues from piglets that had died after the HP-PRRSV challenge were sampled for histological examination. All tissue samples were fixed by immersion in 10% neutral buffered formalin then were typically embedded in paraffin followed by sectioning for use in histological examination. Sections were stained with hematoxylin and eosin (H&E) to detect micropathological changes.

### 2.5. RNA Isolation and Quantitative Real-Time PCR (qPCR)

Total RNA was extracted from serum samples or nasal swabs using TRIzol reagent (Thermo Fisher Scientific, Waltham, MA, USA) in accordance with the manufacturer’s instructions. PRRSV RNA detection via qPCR was conducted using RealPCR PRRSV-2 RNA Mix (IDEXX, Westbrook, ME, USA) following the manufacturer’s instructions. The manufacturer’s cut-off Ct value of 38 was used for analysis of qPCR data that reflected PRRSV RNA levels.

### 2.6. Enzyme-Linked Immunosorbent Assay (ELISA)

Sequential serum samples harvested at indicated time points from all experimental animals were screened for anti-PRRSV antibodies using IDEXX HerdChek PRRS X3 ELISA kit (IDEXX) according to the manufacturer’s instructions.

### 2.7. Virus Neutralization Assay

Virus neutralization assays were carried out using MARC-145 cells and based on the ability of PRRSV-neutralizing antibodies in serum samples to bind to the virus and block infection as previously described [38,39]. Briefly, serum samples were heat-inactivated at 56 ℃ for 30 min followed by 2-fold serial dilutions. To each dilution an equal volume of HP-PRRSV-XJA1 (the same virus used for challenge experiments) was added followed by incubation of the mixtures under 37 ℃ for 1 h to allow antibodies to bind to the virus. After incubation, the mixtures were transferred to MARC-145 monolayers in 96-well plates and incubated for an additional 72 h at 37 ℃ in an incubator with humidified atmosphere containing 5% CO_2_. Cells were examined for cytopathic effects (CPE) and end-point titers were used to calculate serum sample neutralizing antibody titers. 

### 2.8. Statistical Analysis

Results were analyzed using GraphPad Prism version 5.0 (GraphPad Software, San Diego, CA, USA). Statistical significance was determined using either the Student’s *t*-test (for comparisons involving two groups) or one-way analysis of variance (ANOVA; for analysis involving more than two groups). A two-tailed *p* value < 0.05 was considered statistically significant.

## 3. Results

### 3.1. Experimental Design and Immunization Schedules

Previous data had suggested that PRRSV-A2MC2-P90 might function as a vaccine by preventing PRRS after challenge of vaccinated piglets with heterogeneous PRRSV strains (e.g., VR2385, 92% homology to A2MC2-P90), prompting us to evaluate the protective efficacy of PRRSV-A2MC2-P90 in the present study. Briefly, PRRSV-A2MC2-P90 was recovered from infectious clone pBAC-A2MC-P90 that had been generated by the cloning of PRRSV-ACMC2-P90 cDNA into a bacterial artificial chromosome vector functioning as a DNA-launched PRRSV recovery system (data not shown) using a previously described method [32]. After failed attempts to rescue the PRRSV-A2MC2-P90 virus in MARC-145 cells via infectious clone transfection (data not shown), the infectious clone was transfected into three PRRSV-permissive cell lines (CRL-2843^CD163^ cells, PK-15^CD163^ cells and HEK-293T^CD163^ cells) to rescue virus. Viable virus was finally recovered from HEK-293T^CD163^ transfected with the infectious clone (data not shown) and further propagated in MARC-145 cells before it was used to inoculate piglets. Meanwhile, we also evaluated the growth curve (Figure S1A) and IFN inducing capability of recovered virus. Based on our data, supernatant of A2MC2-P90 infection contains a high level of bioactive IFNs, with the highest dilution up to 1:16 in VERO cells against NDV infection (Figure S1B), which was similar to our previous observation [31].

**Figure 1:**
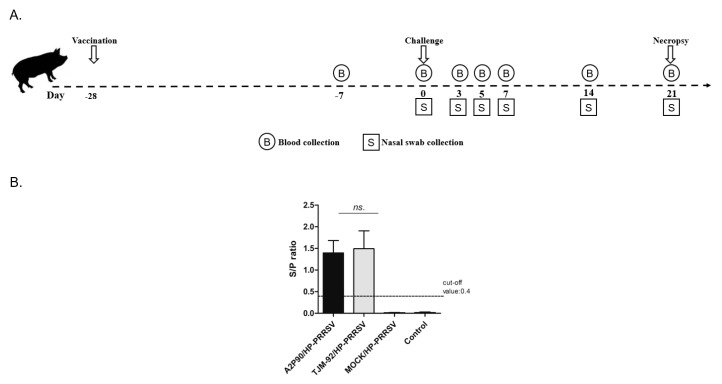
Schematic illustration of experimental protocol and ELISA examination of seroconversion prior to the porcine reproductive and respiratory syndrome virus (PRRSV) challenge. (**A**). After piglets were housed, A2MC2-P90 and TJM-F92 were used to vaccinate animals via the intramuscular route using 1 mL of virus stock (1.0 × 10^6^ TCID_50_/mL). At 21 days post vaccination, serum samples were collected then at day 0 piglets were challenged with HP-PRRSV-XJ1. Blood and nasal swab samples were collected at indicated time points and all surviving animals were necropsied at 21 dpc. (**B**). After immunization of piglets with A2MC2-P90 and TJM-F92, serum samples were collected 21 days later and examined to detect seroconversion using an IDEXX HerdChek PRRS X3 ELISA kit. All experiments were repeated at least three times for each serum sample. Data are expressed as the mean ± SD and were subjected to Student’s *t*-test. No significant differences (NS) were observed between groups immunized with A2MC2-P90 or TJM-F92.

In order to conduct a systematic comparison of protective efficacy, commercially licensed HP-PRRSV MLV strain PRRSV-TJM-F92 and candidate PRRSV-A2MC2-P90 were tested in parallel. The experimental protocol and immunization schedule are illustrated in Figure 1A for the piglet groupings listed in Table 1. During vaccination, the same dose of viral stock (1 mL of 1.0 × 10^6^ TCID_50_/mL) and the same route of administration were used for all piglets, with immunization conducted at −28 dpc. Serum samples from all immunized animals, including PBS-inoculated controls, were collected at 21 days post vaccine immunization or PBS administration (−7 dpc) and subjected to ELISA screening. A serum conversion result (a positive result indicating presence of anti-PRRSV antibodies) was obtained for all vaccinated piglets, with no serum conversion observed for PBS controls (Figure 1B), suggesting that immunizations of piglets with either PRRSV-A2MC2-P90 or PRRSV-TJM-F92 had been successful.

### 3.2. PRRSV-A2MC2-P90 Protected Piglets against a Lethal Challenge with Highly Pathogenic-PRRSV (HP-PRRSV)

**Figure 2:**
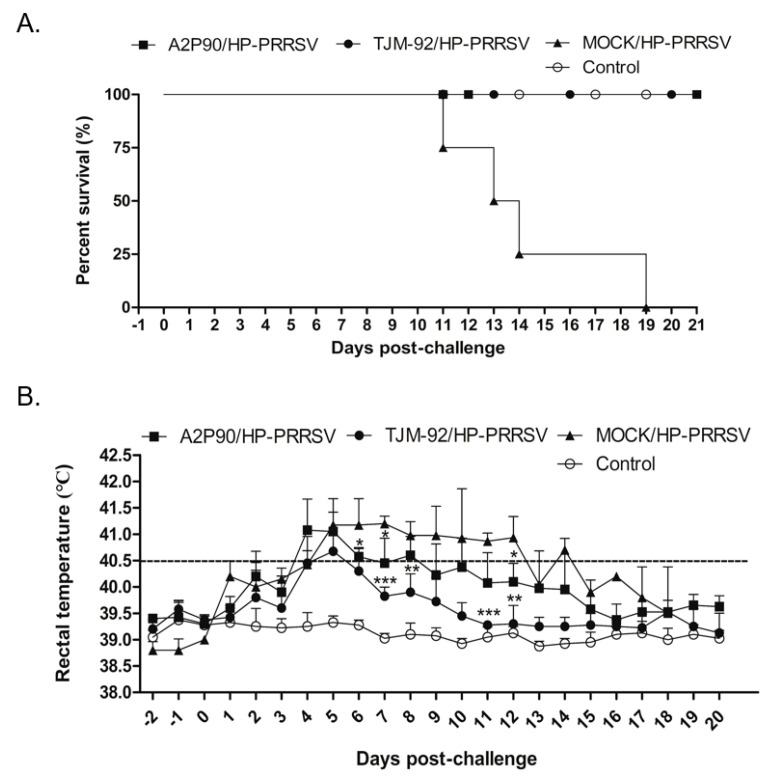
PRRSV-A2MC2-P90 protection of piglets against a lethal challenge with highly pathogenetic PRRSV (HP-PRRSV). (**A**). All vaccinated animals or non-vaccinated controls were each inoculated with 1.0 × 10^5^ TCID_50_ of HP-PRRSV-XJ1 via both intramuscular and intranasal routes. Clinical signs and survival rates were monitored and calculated daily for a total of 21 days. (**B**). After inoculation of animals, rectal temperature was recorded daily for surviving animals of all groups. Data are expressed as mean ± SD and were subjected to a Student’s *t*-test. Significant differences between the A2MC2-P90-vaccinated group (A2P90/HP-PRRSV), TJM F92-vaccinated group (TJM-92/HP-PRRSV) and the non-vaccinated but the HP-PRRSV challenged group (MOCK/HP-PRRSV) are marked with * (*p* < 0.05), or ** (*p* < 0.01) or *** (*p* < 0.001).

After immunization, all vaccinated piglets and a group of non-vaccinated control piglets were inoculated with a highly virulent HP-PRRSV strain XJA1 (HP-PRRSV-XJA1) via both intramuscular and intranasal administration routes to ensure successful infection. Beginning at 11 dpc, mortality due to the HP-PRRSV-XJA1 challenge emerged in the non-vaccinated group and continued to occur until 19 dpc, when the last piglet in this group died (Figure 2A). By contrast, the survival rate was 100% for both A2MC2-P90- and TJM-F92-vaccinated piglets post-HP-PRRSV-XJA1 challenge. Therefore, based on survival rates, A2MC2-P90 and the HP-PRRSV-specific vaccine TJM-F92 conferred the same level of protection against the HP-PRRSV challenge. Meanwhile, to further evaluate clinical signs of piglets in each group, rectal temperatures were recorded daily and compared among groups. In TJM-F92-vaccinated groups, rectal temperatures peaked at 5 dpc then returned to normal by 16 dpc (Figure 2B). By contrast, rectal temperatures reached a peak at 4 dpc then returned to normal by 16 dpc in A2MC2-P90-vaccinated piglets (Figure 2B). Moreover, rectal temperatures of A2MC2-P90-vaccinated piglets at 6, 7, 8, 11 and 12 dpc were statistically significantly higher than corresponding temperatures of TJM-F92-vaccinated piglets. These results suggest that clinical signs of A2MC2-P90-vaccinated piglets were slightly more pronounced than the corresponding signs of piglets in the TJM-F92-vaccinated group; such differences may stem from antigenic variations between A2MC2-P90 (belongs to classical PRRSV, with 99% homology to PRRSV-2 prototype VR2332) and challenging the virus XJA1 strain (belongs to HP-PRRSV, with 88.7% homology to A2MC2-P90 based on GP5 sequence). Nevertheless, these data suggest that A2MC2-P90 provided a level of protection against HP-PRRSV infection that was comparable to that provided by TJM-F92, although A2MC2-P90 is a classical PRRSV isolate.

### 3.3. PRRSV-A2MC2-P90 Vaccination Significantly Alleviated Pathological Lung Lesions after the HP-PRRSV Challenge

**Figure 3:**
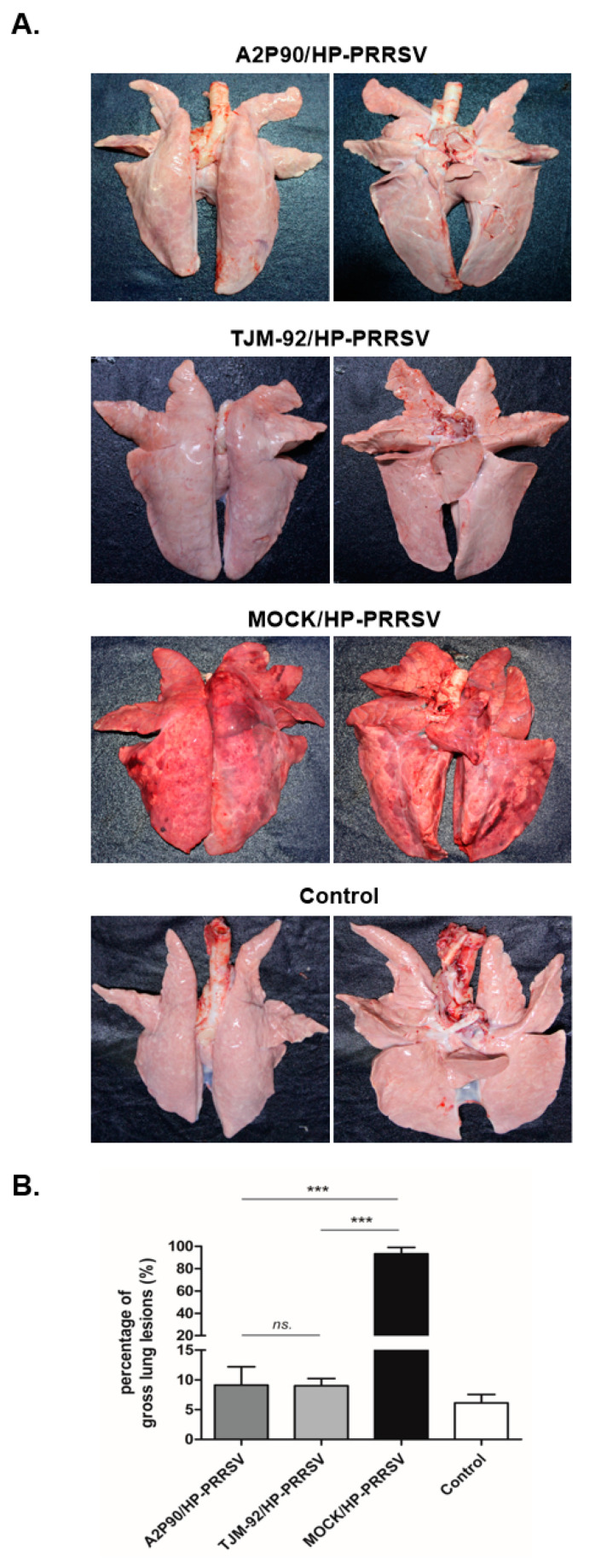
PRRSV-A2MC2-P90 vaccination prevented development of pathological lung lesions after HP-PRRSV challenge in vivo. (**A**). A total of 16 piglets were randomly divided into 4 groups (*n* = 4) that included the negative control group, A2MC2-P90-vaccinated group (A2P90/HP-PRRSV), TJM F92-vaccinated group (TJM-92/HP-PRRSV) and non-vaccinated HP-PRRSV-challenged group (MOCK/HP-PRRSV). Representative ventral and dorsal lung images from each group were captured immediately after piglets were autopsied at 21 dpc or at time of death if before 21 dpc. (**B**). Gross pathological changes of all animals in each group were quantified using a scoring system based on a 100-point scale. Data are expressed as the mean ± SD and were subjected to a Student’s *t*-test. Significant differences between indicated groups were marked with *** (*p* < 0.001) or NS (not significant).

To further understand the pathological changes occurring in each experimental group after the HP-PRRSV challenge, all piglets were autopsied immediately after death or at 21 dpc if they survived until then. As shown in Figure 3A, lung lesions in unvaccinated piglets challenged with HP-PRRSV-XJA1 served as evidence of extensive pneumonia and severe pathological changes associated with the virulent HP-PRRSV-XJA1 challenge. By contrast, lungs of PRRSV-A2MC2-P90- and PRRSV-TJM-F92-vaccinated piglets resembled lungs of uninfected controls. Next, in order to better quantify observed pathological changes, a lung gross lesion score system was employed as previously described [37]. As shown in Figure 3B, lung gross lesion score differences between PRRSV-A2MC2-P90- and PRRSV-TJM-F92-vaccinated groups were not statistically significant. However, in HP-PRRSV-XJA1-inoculated piglets, extremely high scores were obtained relative to scores of other groups. Conversely, a HE stained was conducted for lung tissue samples from the MOCK group (harvested during autopsy 21 dpc) and HP-PRRSV-XJA1-inoculated group (harvested once found dead), demonstrated extensive pneumonia and severe pathological changes due the virulence and pathogenicity of challenged viruses (Figure S2). Collectively, these results demonstrated that prior vaccination with PRRSV-A2MC2-P90 or PRRSV-TJM-F92 significantly prevented pathological lung lesion development in piglets after challenge with HP-PRRSV.

### 3.4. PRRSV-A2MC2-P90 Vaccination Reduced Nasal Virus Shedding and Viremia after HP-PRRSV Challenge

**Figure 4:**
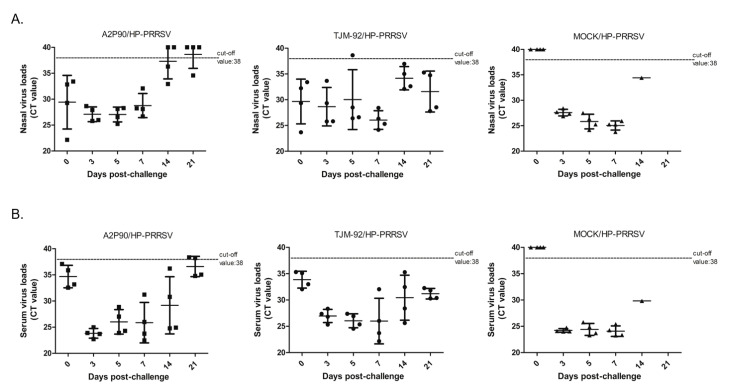
PRRSV-A2MC2-P90 vaccination reduced nasal shedding of virus and viremia after HP-PRRSV challenge of vaccinated piglets. (**A**). Nasal swab samples collected at indicated time points and harvested using TRIzol reagent were subjected to PRRSV RNA detection using IDEXX RealPCR PRRSV-2 RNA Mix. The Ct value of each sample is presented for comparison and was based on the manufacturer cut-off Ct value of 38. (**B**). Serum samples collected from each piglet at indicated time points and processed using TRIzol reagent were subjected to PRRSV RNA detection using IDEXX RealPCR PRRSV-2 RNA Mix. The Ct value of each sample is presented for comparison and was determined based on the manufacturer cut-off the Ct value of 38.

In addition to vaccination effects on survival rates and development of lung lesions, we investigated viral shedding and viremia by measuring PRRSV RNA levels in nasal swabs and blood samples via qPCR analysis. As shown in Figure 4A, PRRSV RNA levels in nasal swabs from PRRSV-A2MC2-P90-vaccinated piglets remained steady from 3 to 5 dpc, with Ct values ranging from 26 to 28. However, starting at 7 dpc, Ct values calculated based on PRRSV RNA levels in nasal swabs exhibited a clearly increasing trend with time (representing reduced viral shedding). In fact, two piglets tested negative for PRRSV RNA in nasal swab at 14 dpc and remained negative through 21 dpc (Figure 4A) and one nasal swab collected from an additional piglet tested negative for PRRSV RNA at 21 dpc. Taken together, these results demonstrate reduced shedding of the virus in PRRSV-A2MC2-P90-vaccinated piglets compared to that of unvaccinated challenged piglets. Conversely, in TJM-F92-vaccinated piglets challenged with HP-PRRSV, all nasal swab samples except for one tested negative for PRRSV RNA at 5 dpc then thereafter no other negative samples were identified for this group. Notably, results for samples harvested at 14 and 21 dpc from TJM-F92-vaccinated piglets after challenge with HP-PRRSV demonstrated that persistent virus shedding occurred at those time points.

PRRSV RNA levels in serum samples followed similar trends as those observed for nasal swabs, with levels in serum samples of PRRSV-A2MC2-P90-vaccinated piglets clearly decreasing after 5 dpc, as reflected by increasing qPCR Ct values. More importantly, two piglets of the A2MC2-P90-vaccinated group were negative for PRRSV RNA based on serum sample results at 21 dpc (Figure 4B), suggesting that a complete clearance of PRRSV viremia occurred in these two animals, while no PRRSV RNA-negative piglets were identified in the TJM-F92-vaccinated group (Figure 4B). It is also notable that although the degree of viremia in TJM-F92-vaccinated piglets at 21 dpc was lower than at 3 dpc, viremia at 21 dpc in TJM-F92-vaccinated piglets still greatly exceeded that of PRRSV-A2MC2-P90-vaccinated piglets, indicating that TJM-F92-vaccinated piglets exhibited delayed reduction of viremia. Therefore, the abovementioned data indicate that PRRSV-A2MC2-P90 immunization reduced virus shedding and viremia after HP-PRRSV challenge, with greater reductions of both indicators observed after PRRSV-TJM-F92 vaccination.

### 3.5. PRRSV-A2MC2-P90 Vaccination Evoked Higher Levels of Neutralizing Antibodies (NAbs)

**Figure 5:**
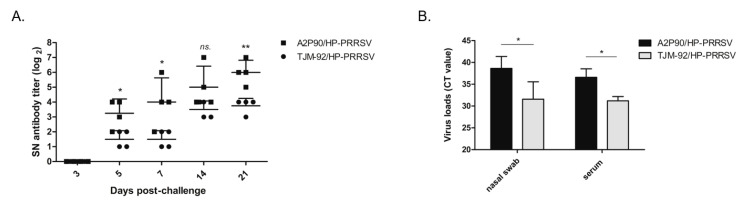
PRRSV-A2MC2-P90 vaccination evoked a higher titer of neutralizing antibodies in vaccinated piglets that correlated with reduced virus shedding and viremia. (**A**). Serum samples from piglets in the PRRSV-A2MC2-P90-vaccinated group (A2P90/HP-PRRSV) or TJM F92-vaccinated group (TJM-92/HP-PRRSV) were collected at indicated dpc after HP-PRRSV challenge. Sera were further tested via neutralizing assays using 2-fold serial dilutions to evaluate virus neutralizing activity against HP-PRRSV infection of MARC-145 cells. Data are expressed as the mean ± SD and were subjected to a Student’s *t*-test. Significant differences of NAbs titers between A2P90/HP-PRRSV and TJM-92/HP-PRRSV at indicated dpc (except 3 dpc) were marked with * (*p* < 0.05), or ** (*p* < 0.01) or NS (not significant). (**B**). Nasal swabs and serum samples from both vaccination groups collected at 21 dpc were processed using TRIzol reagent and subjected to PRRSV RNA detection using IDEXX RealPCR PRRSV-2 RNA Mix. The Ct value of each sample is presented as the mean ± SD and all data were subjected to a Student’s *t*-test. Significant differences of Ct values between the A2MC2-P90-vaccinated group (A2P90/HP-PRRSV) and TJM F92-vaccinated group (TJM-92/HP-PRRSV) are marked with * (*p* < 0.05).

In our previous investigation, intramuscular and intranasal immunization of piglets with A2MC2, the parental strain of A2MC2-P90 (with a moderately virulent phenotype), induced much higher titers of NAbs starting from 28 dpi as compared to titers in piglets inoculated with VR2385 and MLV strains. Moreover, NAbs titers of A2MC2-inoculated piglets continually increased until the end of the study at 56 dpi. [24]. However, it remained to be investigated whether A2MC2-P90 would also elicit high titers of NAbs. Therefore, we conducted neutralization assays to test for the presence of PRRSV-specific NAbs in serum samples of both vaccinated groups against HP-PRRSV-XJA1 (the same virus used for challenge). As demonstrated in Figure 5A, before 0 dpc (28 days after vaccination), serum NAbs levels were very low in all piglets regardless of PRRSV strain used for immunization. This changed beginning at 5 dpc (33 days after vaccination), when NAbs titers of the HP-PRRSV-challenged PRRSV-A2MC2-P90-vaccinated group (designated A2P90/HP-PRRSV) became significantly higher than the TJM-F92 group and remained at a much higher level until study completion at 21 dpc (49 days after vaccination; Figure 5A). Although no significant differences in NAbs titers were observed at 14 dpc between the two vaccinated groups of piglets, clearly higher NAbs titers were observed in HP-PRRSV-challenged A2MC2-P90-vaccinated piglets versus HP-PRRSV-challenged TJM-F92-vaccinated piglets. Moreover, NAbs titers of the TJM-F92 vaccination group did not markedly change between 14 dpc and 21 dpc, while NAbs titers in the A2MC2-90-vaccinated group continually increased in the same period (Figure 5A). When considered together, these results suggest that A2MC2-90 induced higher levels of NAbs, which appears to be similar of previous observation in its parental strain A2MC2 as previously reported [24].

Previous reports investigating PRRSV-specific NAbs kinetics have suggested that the length of time between experimental infection and onset of NAbs detection correlates with virus clearance from circulation and tissues [14,40]. Therefore, a statistical analysis was conducted here to correlate NAbs titer with viremia or nasal virus shedding in our challenging experiments. On the one hand, as shown in Figure 5A, a statistically higher level of NAbs in the A2MC2-90-vaccinated group was observed as compared with the TJM-F92-vaccinated group. On the other hand, as shown in Figure 5B, viremia and nasal virus shedding levels (data presented as qPCR Ct values) of A2MC2-90-vaccinated piglets were statistically much lower than respective levels observed for the TJM-F92-vaccinated group, with three and two A2MC2-90-vaccinated piglets testing negative for PRRSV RNA in nasal swabs and serum samples, respectively, as based on the manufacturer’s cut-off Ct value. Taken together, these data further confirmed that higher NAbs titers contributed to more rapid clearance of viremia and reduced virus shedding in piglets.

In conclusion, our data suggest that A2MC2-P90 vaccination protected 100% of piglets from heterogeneous challenging with HP-PRRSV strain XJA1 and also provided comparable efficacy to that of the commercially licensed HP-PRRSV vaccine. Notably, A2MC2-P90-immunized piglets mounted a more rapid protective humoral immune response that was characterized by a much higher neutralizing antibodies titer, more rapid clearance of viremia and less virus shedding than elicited by vaccination with the current HP-PRRSV vaccine. Therefore, PRRSV-A2MC2-P90 shows promise as a potential vaccine that appears to provide a high degree of protection against PRRSV infection of swine across an improved protection spectrum of heterogeneous PRRSV strains.

## 4. Discussion

In 2007, the Colloquium on Prospects for Development of an Effective PRRSV Virus Vaccine was held at the College of Veterinary Medicine, University of Illinois, in the United States to discuss the state of current knowledge regarding PRRS vaccination [41]. All attendees, including experts in PRRS, virology, immunology and vaccinology, as well as clinical veterinarians, academics and vaccine industry scientists, set new standards for the next generation of PRRSV vaccines. These standards included requirements that candidate vaccines elicit rapid induction of immunity, protect against most currently prevalent PRRSV strains, do not adversely affect swine health and possess features that will enable differentiation between vaccinated and naturally infected animals [41]. However, in spite of the fact that over ten years have passed since this colloquium, no vaccine candidates meeting all of the abovementioned criteria are commercially available.

Inhibition of innate immunity by PRRSV infection was considered as a major factor contributing to PRRSV pathogenesis. Type I interferons (IFNs), including IFN-α, IFN-β, IFN-ε and IFN-κ, comprise of the largest family of IFNs and play major roles in innate immunity against viral infections [42,43]. Induction of IFNs typically results from activation of host pattern-recognition receptors (PRRs), such as RIG-I-like receptors (RLR) and toll-like receptors (TLR) [44]. Cell stimulation by IFNs involves activation of the JAK/STAT pathway [45], which leads to expression of IFN-stimulated genes (ISGs) that act as antiviral effectors to restrict virus replication. Meanwhile, IFNs also exhibit antiproliferative activity, stimulate cytotoxic T cells and modulate immune responses [45]. Intriguingly, the PRRSV genome encodes several IFNs antagonists that block both IFN induction and IFN-activated JAK/STAT signaling [46,47,48]. PRRSV non-structural proteins (nsp1α, β and 2) inhibit IFN-β expression through effects on the IRF3 signaling pathway [49,50,51], while PRRSV-nsp4 interferes with the NF-κB signaling pathway via cleavage of NEMO [52] and PRRSV-nsp11 suppresses transcriptional activation of IFN-β via endoribonuclease-mediated cleavage of MAVS mRNA [53]. Meanwhile, PRRSV nsp1β also inhibits IFN-activated JAK/STAT signaling by inducing degradation of KPNA1, a critical transporter protein that mediates nuclear import of ISGF3 [46,54]. Although in vivo studies have suggested that certain PRRSV isolates (e.g., HP-PRRSV HuN4-F112) could induce IFN-α secretion [55,56,57] but still blocking the IFN-activated JAK/STAT pathway, low levels of bioactive IFN-α may not be sufficient to activate the antiviral response [46,48]. Furthermore, although IFNs induction typically results from activation of PRRs, IFNs are not the only cytokines produced after PRRs activation [44]. Therefore, IFNs acting alone might not be capable of activating host innate immunity, prompting speculation that a synergistic effect mediated by both IFNs and other proinflammatory cytokines may be required in order to fully elicit immune responses in PRRSV-infected hosts.

PRRSV-A2MC2, the first reported novel PRRSV strain to elicit strong IFN synthesis in cultured cells, shares the highest nucleotide identity with Ingelvac PRRS^®^MLV and its prototype VR-2332 [24,29]. It is notable that the first 4.6 kb at the 5′ primer end of the A2MC2 genome (including coding regions for putative IFNs antagonists nsp1α, nsp1β and nsp2) is identical to the corresponding genomic region of VR-2332, which exhibits an IFN-inhibitory phenotype. This observation, coupled with results of reverse genetics-based gene fragment swapping experiments, have provided information that roughly pinpoints the location of the genomic region responsible for IFN induction to within the middle half of the A2MC2 genome [58]. Notably, the attenuated A2MC2-P90 strain maintains an IFNs-inducing phenotype while also protecting piglets from heterogeneous PRRSV challenge even though this strain is avirulent MLVs [31]. Nevertheless, in spite of its avirulent phenotype and the fact that it belongs to the classical group of PRRSV strains sharing 99% homology to VR2332 (PRRSV-2 prototype), here A2MC2-P90 immunization conferred effective protection against extremely virulent HP-PRRSV (sharing 88.7% homology to A2MC2-P90 and VR2332) and had protective efficacy comparable to that of commercial HP-PRRSV-specific vaccine TJM-F92. Thus, these data collectively demonstrate that A2MC2-P90 conferred improved protection spectrum against heterogeneous PRRSV challenge and thus this vaccine candidate holds promise as an effective MLV.

Aside from its safety and efficacy, another novel characteristic of A2MC2-P90 is its ability to elicit high titers of PRRSV-specific neutralizing antibodies (NAbs) when used to vaccinate piglets. In an early report, single dose immunization of piglets with the parental strain A2MC2 elicited NAbs production of earlier onset that generated higher titers of neutralizing antibodies against homologous and heterologous strains than NAbs levels induced in PRRSV-VR2385- or Ingelvac PRRS^®^MLV-vaccinated piglets [24]. Notably, ACMC2-P90-vaccinated piglets generated similar NAbs titers as observed for piglets vaccinated with its unattenuated parental strain AC2MC2 or with TJM-F92 during the first 4 weeks after vaccination but prior to challenge with virulent virus [24]. However, during the HP-PRRSV-XJA1 challenge, (except at 14 dpc), serum NAbs titers of A2MC2-P90-vaccinated piglets were significantly higher than titers of TJM-F92 vaccinated-piglets, with NAbs titers continually increasing from 5 dpc until the end of the experiment at 21 dpc. Tentatively, these limited data align with early and enhanced production of NAbs induced by A2MC2 immunization of piglets as reported previously [24], suggesting that attenuation of virulence did not affect NAbs induction characteristics of A2MC2-P90 in piglets. Importantly, here we must note that groups immunized with A2MC2-P90 were not included here as a non-challenge control; thus, it is not certain whether the HP-PRRSV-JXA1 challenge actually contributed to the marked elevation of Nabs titers in A2MC2-P90-vaccinated piglets by further boosting the immune response induced earlier by A2MC2-P90 vaccination but requires further investigation. Therefore, these data implies that A2MC2-P90 immunization induced a protective immune response that included higher NAbs titers than those generated by conventional vaccination with PRRSV-MLVs such as TJM-F92, even after challenge with a heterogeneous virus. It is not known whether the higher NAbs titers observed in A2MC2-P90-immunized piglets was a direct consequence of the IFN-inducing phenotype of A2MC2-P90, highlighting the need for further investigations to reveal a link between IFNs induction and activation of the humoral immune response in swine species. Indeed, such work would likely provide new insights to guide future vaccine development against swine pathogens.

In recent years, researchers have considered PRRSV-specific NAbs to be an essential component of protective immunity against PRRSV [20,59], as killed virus vaccines (KIV) have only been shown to induce non-protective PRRSV-specific NAbs in vaccinated piglets [20,59]. Notably, results of kinetic studies of PRRSV-specific NAbs production have suggested that the duration of time between experimental infection and onset of NAbs production correlates with virus clearance from the circulation and from tissues. Meanwhile, other studies have shown that passive transfer of PRRSV-specific NAbs protected pregnant sows against reproductive failure and induced sterilizing immunity in herds and offspring in a dose-dependent manner [60]. Taken together, these results indicate that maintaining a high level of PRRSV-specific NAbs in vaccinated piglets is crucial for the development of herd immunity. However, during the course of natural PRRSV infection in pigs or after PRRSV-MLVs vaccination, PRRSV-specific NAbs do not typically appear until 28 days post-inoculation and thereafter persist only at a relatively low level for months [59,61]. Nevertheless, the duration of the aforementioned PRRSV NAbs production is comparatively longer that of porcine epidemic diarrhea virus (PEDV), another member of *Nidovirales*, which induces neutralizing antibodies within two weeks of infection [19]. Notably, a vaccine’s ability to induce the production of a high titer of NAbs may be associated with the vaccine’s ability to achieve superior herd immunity, which has not been achieved through vaccination with conventional MLVs. Moreover, this concept also aligns with our results showing that serum PRRSV RNA levels of A2MC2-P90-immunized piglets after HP-PRRSV challenge were significantly lower than corresponding PRRSV RNA levels in the serum of piglets in the challenged TJM-F92-immunized group. It is also notable that two piglets from the challenged A2MC2-P90-immunized group reverted to PRRSV RNA-negative status at 21 dpc, suggesting that complete clearance of PRRSV viremia had occurred in these animals, while no serum PRRSV RNA-negative piglets were identified in the other experimental groups. These data imply that A2MC2-P90 vaccination of piglets led to more rapid clearance of viremia relative to that of the TJM-F92-vaccinated group, a result that may stem from higher serum NAbs titers in the challenged A2MC2-P90-vaccinated group.

In addition to low levels of viremia, examination of PRRSV RNA isolated from nasal swabs from two piglets in the A2MC2-P90-immunized group indicated they stopped shedding virus, as also evidenced by negative PRRSV RNA detection results in nasal swabs at 14 dpc and an additional animal in that group was PRRSV RNA negative in a nasal swab at 21 dpc. Taken together, a clear trend of more efficient virus clearance and less virus shedding was observed in the A2MC2-P90-immunized group, as consistent with higher serum NAbs titers. By contrast, no serum or nasal swab samples taken from the TJM-F92 group was negative for PRRSV RNA at any time points throughout the study. Therefore, A2MC2-P90 immunization elicited a rapid and higher NAbs response that was sustained during heterogeneous PRRSV wild-type virus challenge. It would thus be interesting to know if the higher NAbs titers in the sera of this group would persist in the long-term to confer longer protection against PRRSV infection than is achievable using the current TJM-F92 vaccine.

In conclusion, our data suggest that the A2MC2-P90 vaccination of piglets conferred 100% protection from infection after challenge with extremely virulent HP-PRRSV strain XJA1. Notably, A2MC2-P90-immunized piglets mounted a rapid protective humoral immune response characterized by a very high titer of neutralizing antibodies, rapid clearance of viremia and a low level of virus shedding. Taken together, our data suggest that A2MC2-P90 is a novel PRRSV vaccine candidate that may confer broad improved protection spectrum against heterogeneous PRRSV strains.

## Figures and Tables

**Table 1:** Animal groups.

Group Name	Vaccine Immunized	Virus Challenged
Control	PBS	PBS
MOCK/HP-PRRSV	PBS	PRRSV-XJA1
A2P90/HP-PRRSV	A2MC2-P90	PRRSV-XJA1
TJM-92/HP-PRRSV	TJM-92	PRRSV-XJA1

## Supplementary Materials

The following are available online at https://www.mdpi.com/2076-393X/8/3/490/s1, Figure S1: Evaluation of PRRSV-A2MC2-P90 recovered from pBAC-A2P90. A. Multi-step growth curve of A2MC2-P90 in MARC-145 cells. The cells were inoculated with A2MC2-P90 virus at a 1 multiplicity of infection (MOI). Virus yields at different time points after inoculation were titrated using MARC-145 cells. Error bars represent variation of three repeated experiments. B. Interferon bioassay in Vero cells. Dilutions of cell culture supernatant of MARC-145 cells infected with A2MC2-P90 were used to treat VERO cells. Treatment with 10 ng human IFN-α2b was included as a control. At 24 h after the treatment the VERO cells were inoculated with NDV-GFP. At 24 h post-inoculation of NDV, the cells were observed under fluorescence microscopy, Figure S2: HE stains of lung tissue section from MOCK group and autopsied piglets in HP-PRRSV challenged group.
