# Supplementary material for: Interferon Inducing Porcine Reproductive and Respiratory Syndrome Virus Vaccine Candidate Protected Piglets from HP-PRRSV Challenge and Evoke a Higher Level of Neutralizing Antibodies Response
Source: Vaccines (Basel). 2020 Aug 31;8(3):490. doi: 10.3390/vaccines8030490 (PMC7565719; doi:10.3390/vaccines8030490)
Supplement: Supplementary file 1 [file vaccines-08-00490-s001.pdf]

vaccines

MDPI

Article

Interferon Inducing Porcine Reproductive and Respiratory Syndrome Virus Vaccine Candidate Protected Piglets from HP-PRRSV Challenge and Evoke a Higher Level of Neutralizing Antibodies Response

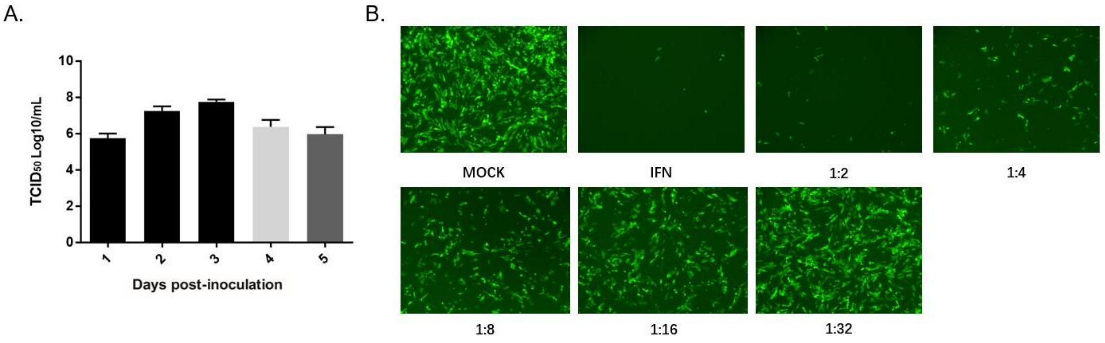

Figure S1. Evaluation of PRRSV-A2MC2-P90 recovered from pBAC-A2P90. A. Multi-step growth curve of A2MC2-P90 in MARC-145 cells. The cells were inoculated with A2MC2-P90 virus at a 1 multiplicity of infection (MOI). Virus yields at different time points after inoculation were titrated using MARC-145 cells. Error bars represent variation of three repeated experiments. B. Interferon bioassay in Vero cells. Dilutions of cell culture supernatant of MARC-145 cells infected with A2MC2-P90 were used to treat VERO cells. Treatment with 10 ng human IFN-α2b was included as a control. At 24 h after the treatment the VERO cells were inoculated with NDV-GFP. At 24 h post-inoculation of NDV, the cells were observed under fluorescence microscopy.

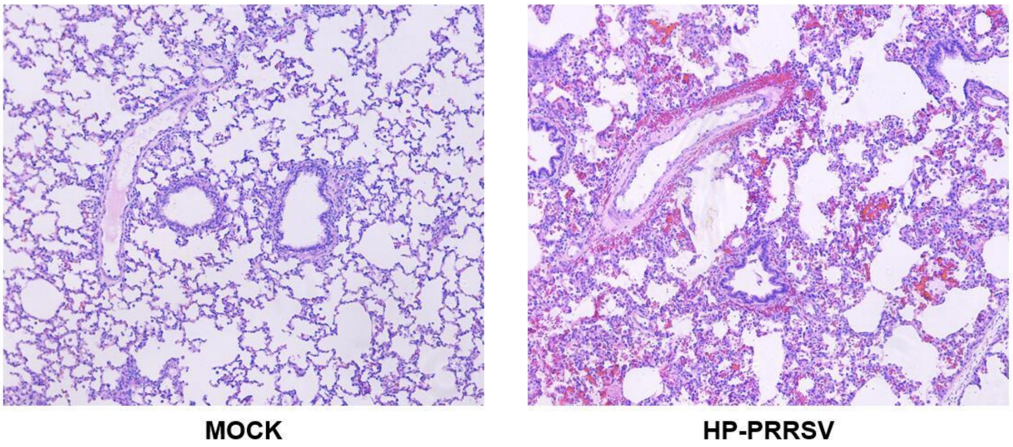

Figure S2. HE stains of lung tissue section from MOCK group and autopsied piglets in HP-PRRSV challenged group.

© 2020 by the authors. Submitted for possible open access publication under the terms and conditions of the Creative Commons Attribution (CC BY) license (http://creativecommons.org/licenses/by/4.0/).
